# Accuracy of detection of carboxyhemoglobin and methemoglobin in human and bovine blood with an inexpensive, pocket-size infrared scanner

**DOI:** 10.1371/journal.pone.0193891

**Published:** 2018-03-07

**Authors:** Margot P. Bickler, Laura J. Rhodes

**Affiliations:** Department of Anesthesia and Perioperative Care, Hypoxia Research Laboratory, University of California, San Francisco, California, United States of America; Consiglio Nazionale delle Ricerche, ITALY

## Abstract

Detecting life-threatening common dyshemoglobins such as carboxyhemoglobin (COHb, resulting from carbon monoxide poisoning) or methemoglobin (MetHb, caused by exposure to nitrates) typically requires a laboratory CO-oximeter. Because of cost, these spectrophotometer-based instrument are often inaccessible in resource-poor settings. The aim of this study was to determine if an inexpensive pocket infrared spectrometer and smartphone (SCiO^®^Pocket Molecular Sensor, Consumer Physics Ltd., Israel) accurately detects COHb and MetHb in single drops of blood. COHb was created by adding carbon monoxide gas to syringes of heparinized blood human or cow blood. In separate syringes, MetHb was produced by addition of sodium nitrite solution. After incubation and mixing, fractional concentrations of COHb or MetHb were measured using a Radiometer ABL-90 Flex® CO-oximeter. Fifty microliters of the sample were then placed on a microscope slide, a cover slip applied and scanned with the SCiO spectrometer. The spectrograms were used to create simple linear models predicting [COHb] or [MetHb] based on spectrogram maxima, minima and isobestic wavelengths. Our model predicted clinically significant carbon monoxide poisoning (COHb ≥15%) with a sensitivity of 93% and specificity of 88% (regression r^2^ = 0.63, slope P<0.0001), with a mean bias of 0.11% and an RMS error of 21%. Methemoglobinemia severe enough to cause symptoms (>20% MetHb) was detected with a sensitivity of 100% and specificity of 71% (regression r^2^ = 0.92, slope P<0.001) mean bias 2.7% and RMS error 21%. Although not as precise as a laboratory CO-oximeter, an inexpensive pocket-sized infrared scanner/smartphone detects >15% COHb or >20% MetHb on a single drop of blood with enough accuracy to be useful as an initial clinical screening. The SCiO and similar relatively low cost spectrometers could be developed as inexpensive diagnostic tools for developing countries.

## Introduction

Acquired dyshemoglobinemias, such as those caused by carbon monoxide poisoning (resulting in carboxyhemoglobin formation, COHb) and nitrate poisoning (causing methemoglobin formation, MetHb) are relatively common, but often difficult to diagnose in low and middle income countries. Accurate diagnosis typically requires both a high index of clinical suspicion; e.g. a history of known exposure to carbon monoxide, industrial chemicals, nitrate fertilizers, etc., and analysis of a blood sample in a laboratory CO-oximeter[1–3]. However, CO-oximeters are expensive and often inaccessible in resource poor settings.

Dyshemoglobinemias can cause life-threatening impairment of blood oxygen delivery to tissues. Carbon monoxide prevents oxygen binding to hemoglobin and also induces a left-shift in the oxygen binding curve, producing impaired oxygen off-loading in tissue and tissue hypoxia [2]. Carbon monoxide poisoning may cause up to 40,000 deaths and >500,000 illnesses per year worldwide[4]. The majority of cases occur in low and middle income countries [5], most frequently due to improperly vented combustion sources. MetHb is a dysfunctional hemoglobin that prevents normal O_2_ binding to hemoglobin because the heme iron atom in MetHb is in the ferric state (Fe^+3^) rather than the normal ferrous (Fe^+2^). Common MetHb-inducing compounds include nitrate fertilizers, pesticides such as propanil, nitric oxide gas, sodium nitrite used for meat preservation, the blood pressure medication sodium nitroprusside, the antibiotic dapsone and the local anesthetic benzocaine[6]. Methemoglobinemia also results from one of several genetic defects in genes coding for proteins that regulate the oxidation-reduction state of heme iron, such as NADH-diaphorase[7, 8]. Individuals with these genetic conditions can have blood metHb levels >30% and purplish skin discoloration [9]. The incidence of methemoglobinemia is not known.

The current standard for measuring the presence of carboxyhemoglobin or methemoglobin in blood is a laboratory CO-oximeter or hemoximeter[10]. These instruments are expensive (typically >$20,000) and require costly calibration and standardization reagents, making them cost-prohibitive in resource-poor settings. More recently, Masimo Inc. (Irvine CA, USA) has introduced a line of pulse CO-oximeters (Rainbow^®^) that non-invasively estimate COHb and MetHb with good sensitivity and specificity[11–15]. Pulse CO-oximeters are also expensive, with instruments costing >$5000 and limited-use probes costing >$100 each.

**Fig 1:**
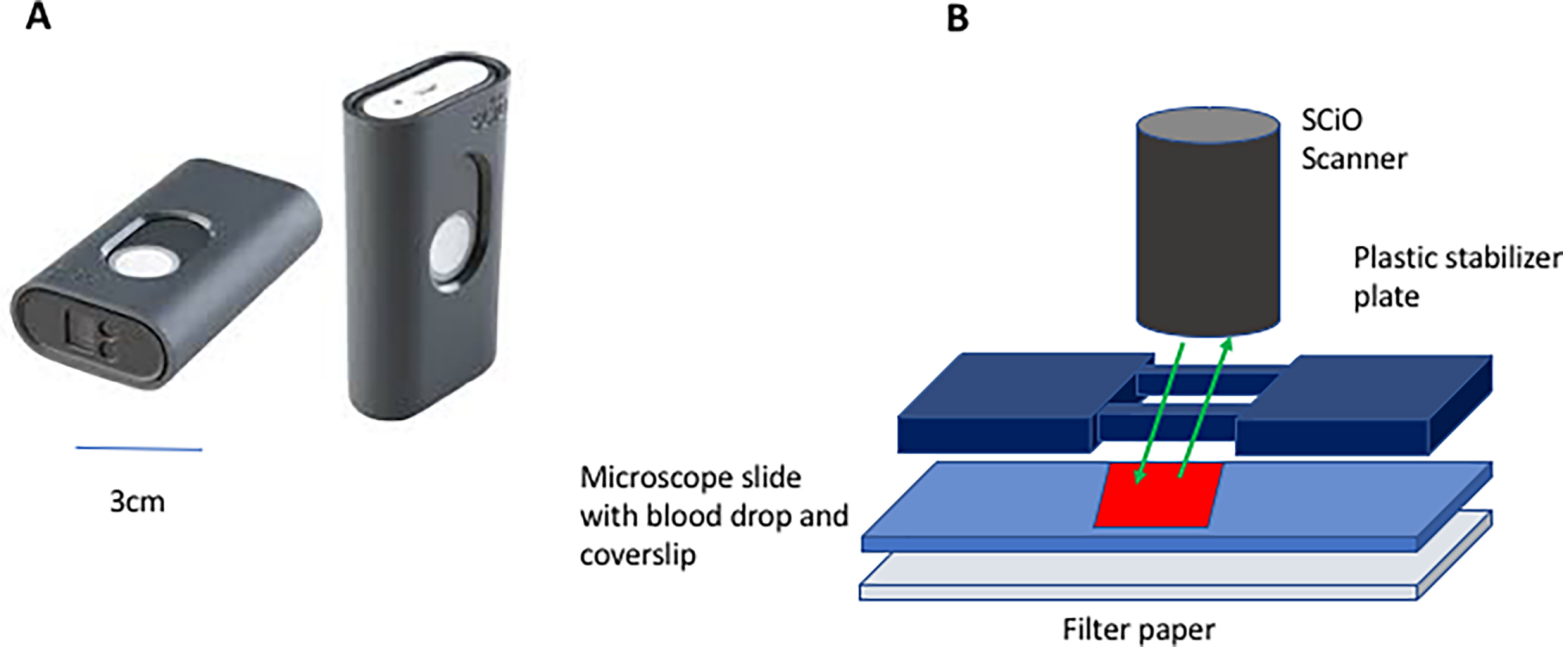
The SCiO (Consumer Physics Ltd., Israel) pocket infrared scanner (Panel A) and schematic diagram of the device in position for scanning a 2.5 cm x 7.5 cm microscope slide with a coverslip and a drop of blood (Panel B).

In this study we tested a pocket-sized infrared spectrometer/scanner’s capability of detecting dyshemoglobin in blood, knowing that abnormal hemoglobin has a unique visible and infrared light reflection spectrum [16, 17]. The device we used, the SCiO pocket molecular scanner (Consumer Physics, Israel), emits infrared light from ~750 nm to 1050 nm and contains an infrared detector (Fig 1A). The device is marketed to consumers and businesses to determine food quality and composition or to identify the chemical composition of medications, etc. Using free software and a smartphone, SCiO users acquire infrared scans, and use an internet-based algorithm to create models that predict the composition of unknown samples. For our analysis, we used the device in the raw spectrogram mode.

## Materials and methods

### Production and measurement of carboxyhemoglobin in cow and human blood *in vitro*

Hemoglobin has an affinity for carbon monoxide 250 times greater than for oxygen [18]. Known volumes of pure CO gas were added to syringes of heparinized human or cow blood to produce COHb levels of 0.5% to 90%. An additional satisfactory method was mixing known quantities of 95% COHb blood with normal blood to achieve desired levels of COHb. Bovine blood was obtained from Lampire Biological Laboratories (Pipersville, PA, adult cow blood in Alsever’s anticoagulant solution, cat # 7200803) and fresh human blood was obtained after written informed consent from healthy volunteer donors. The protocol was approved by the UCSF Committee on Human Research, Protocol No. 10–03634. Human blood was anti-coagulated by addition of sodium heparin to the collection syringe.

### Methemoglobin

Sodium nitrite (20 mg/ml NaNO_3_ in saline) solution was added to bovine or human blood to create metHb. Sodium nitrite induces methemoglobin by removing an electron from the ferrous iron (Fe^+2^) in hemoglobin, converting the heme iron to the ferric state (Fe^+3^). Samples were mixed and held at 37 degrees C for 20 minutes and then transferred to ice until analysis.

### CO-Oximetry

Each blood sample was analyzed in a Radiometer ABL-90 Flex CO-oximeter (Radiometer, Copenhagen DK), calibrated with internal reference standards. This instrument is a hospital clinical laboratory grade self-calibrating CO-oximeter with accuracy of detection of metHb and COHb of +/- 0.5–1%, depending on range, and is considered a gold-standard for MetHb and COHb measurement. The hemoximeter was maintained under service contract by Radiometer America and was current with software updates through September 2017.

### Generation of SCiO spectrograms

For scanning with the SCiO spectrometer, 50μl of blood was placed on a microscope slide and a cover slip was added. The slide was placed on a white filter paper and the scanner was positioned over the slide using a machined gray plastic stand to stabilize the scanner (Fig 1B). Each blood sample was scanned in triplicate using the SCiO Lab App on an Apple iPhone 6, generating spectrograms for each. The SCiO app also is available for all smartphones.

The raw signals from the SCiO were then inspected to determine reflectance signal peaks, minima, and isosbestic wavelengths (R_isobestic_). Simple linear models were used to describe spectrogram data based on the formula

$$[{Hb}_{dys}]=f(R_{\lambda 1}-R_{\lambda 2})/R_{isobestic}$$

where *R*_*λ*1_ and *R*_*λ*2_ are the reflectance spectrogram signals at signal maxima or minima. Simple linear regression analysis of predicted metHb and COHb levels were found to yield regressions with significant slopes and correlation coefficients (r^2^) greater than 0.4. We did not pursue more complex modeling because we wanted to validate a method that would enable users to easily obtain information from spectrograms that could predict COHb or MetHb levels.

### Descriptive statistics

Regression lines and correlation coefficients were calculated by standard methods. Bias was calculated as the SCiO estimated COHb or metHb minus the corresponding hemoximeter reading. The standard deviation of the bias, or the precision, was also calculated. Root mean square error of the bias was calculated as:

$$RMS\;error=\sqrt{({\left(SCio\;estimated\;[COHb\;or\;metHb]-hemoximeter\;measured\;[COHb\;or\;metHb]\right)}^{2}}/N))$$

### Sensitivity and specificity analysis

Studies in healthy human volunteers demonstrate that ~15% COHb and MetHb levels are tolerated by healthy humans without headache, nausea, decreases in blood pressure, or increases in heart rate [12, 13]. At levels above 15% COHb, humans develop nausea, headache and malaise, and at levels of 30% or greater, hemodynamic instability and mortality can occur[2, 19]. For MetHb, patients with chronically elevated MetHb levels from genetic defects for example, may tolerate 30% metHb due to long-term adaptation to this condition. However, acute increases in MetHb to above 20% can produce fatigue, acidosis and decreases in blood pressure [6, 19]. Although the tolerable limits of acute increases in MetHb are not known, levels of >20% metHb are expected to be clinically important in any situation. Therefore, cutoffs of 15% COHb and 20% MetHb were therefore of primary interest in the sensitivity and specificity analysis. We calculated positive predictive value, negative predictive value, sensitivity, and specifity based on a range of cut-off levels for both COHb and MetHb using 15% 20% as the primary cut-off. In addition, the area under the receiver operating characteristic (ROC) curve was determined for the SCiO’s estimates of [COHb] and [MetHb], based on cutoffs of ≥15% for COHb and ≥20% for MetHb. Statistical analysis was performed with Prism 7 (GraphPad Software, Inc., San Diego, CA).

## Results

### Detection of carboxyhemoglobin in human and cow blood

**Fig 2:**
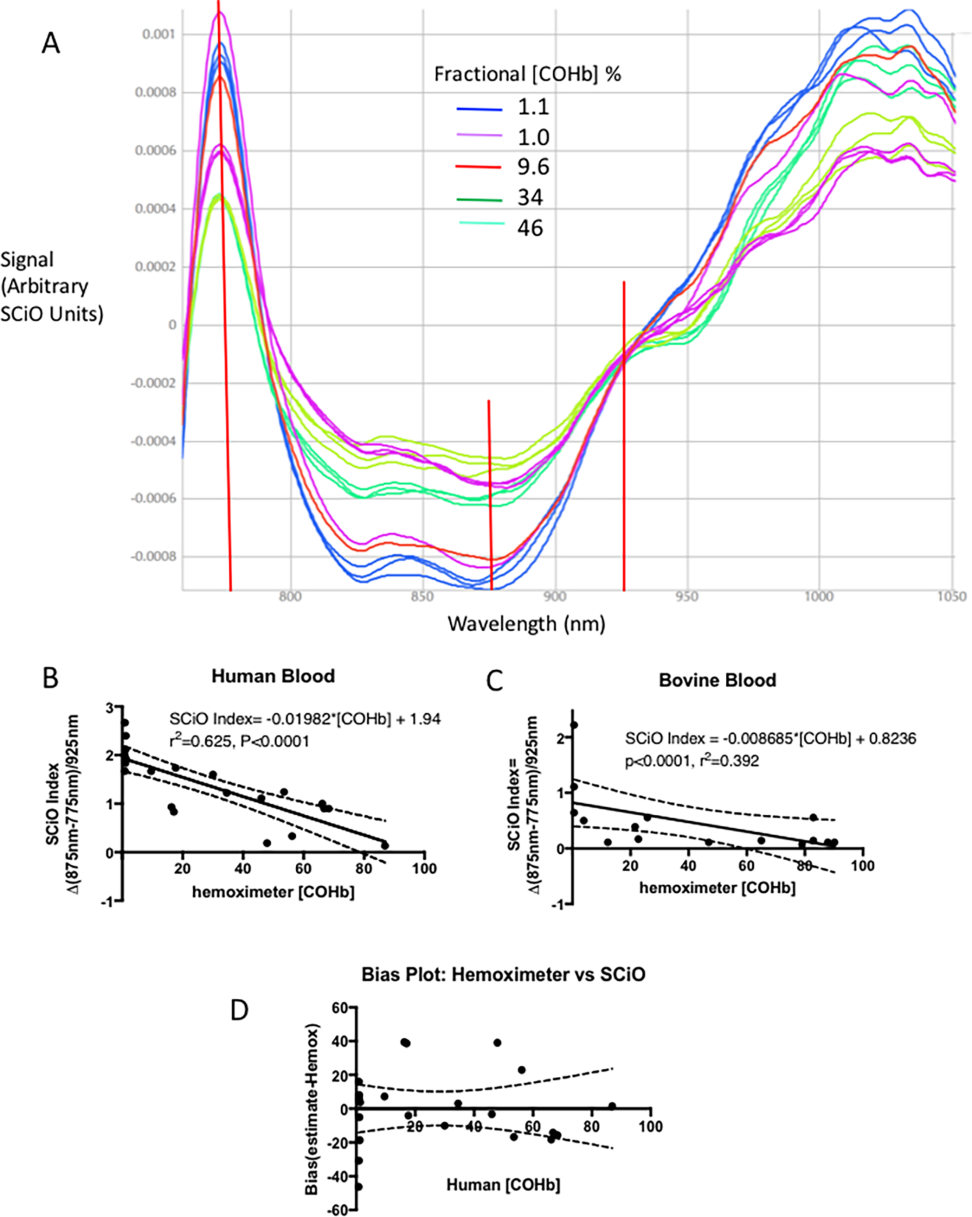
Detection of carboxyhemoglobin (COHb) in thin films of 50 μL of human and bovine blood *in vitro* with spectrograms from the SCiO pocket molecular sensor. **A**. Representative spectrograms of human blood with varying fractional content of COHb, produced by adding pure CO gas to heparinized blood. Hemoximeter measured COHb is indicated, expressed as % of total hemoglobin, as are the wavelengths of interest in the analysis (775 nm, 875 nm and 925 nm). The multiple traces of the same color indicate repeat scans of the same sample, to indicate reproducibility. **B**. Regression analysis of spectrometric model [COHb] = f(850nm spectrogram signal-775 nm spectrogram signal)/925 nm signal and hemoximeter measured COHb levels in human and **C**. bovine blood, based on creation of an index relating differences in spectrogram signals at 875 and 775 nm relative to an isobestic wavelength of 925 nm (see text). **D**. Modified Bland-Altman bias plot of errors in SCiO estimates of [COHb] % based on the regression equation relating SCiO index value and hemoximeter measured [COHb]. Dashed lines show 95% confidence limits for the regressions.

SCiO spectrograms on blood containing elevated COHb were measured in triplicate from 18 bovine and 22 human samples. Each sample was mixed with known amounts of pure CO gas, creating a range of [COHb] from 0.6% (normal for non-smokers) to 87.9% (well above lethal). Spectra from cow blood and human blood with normal and increased [COHb] consistenly revealed an isobestic wavelength of ~925 nm, i.e. the spectrogram signal at 925 nm was independent of [COHb]. Representative SCiO generated spectrograms for human blood containing different amounts of COHb are shown in Fig 2. A peak in signal consistently was observed at ~775 nm for blood containing <10% COHb, whereas blood containing abnormally high levels of COHb had lower spectrogram readings at that wavelength. In addition, a flattening, of the spectrogram curve was observed at ~875 nm for blood containing higher (>15%) COHb levels. Expressing the differences in 875–775 signal as a ratio with the isobestic wavelength of 925 nm created a simple linear model to describe the relationship between SCiO readings and [COHb]:

[COHb] = f(850nm spectrogram signal-775 nm spectrogram signal)/925 nm signal

We termed the spectrogram signal ratio the “SCiO index”.

The slope of the relationship between SCiO and hemoximeter measured COHb was significantly different from zero (P<0.0001, Fig 2, Panels B and C). Using the equation of the linear models, we calculated the predicted [COHb] from the SCiO index readings. The bias of these readings, combining both human and bovine blood, as a function of true [COHb] are shown in a modified Bland-Altman bias plot in Fig 2, Panel D. The mean bias of the estimated [COHb] was 0.108%, with the precision (standard deviation of the bias) 22% and the RMS error 21% for this model. Reproducibility of scans done repeatedly on the same blood sample was high, with spectrogram readings typically varying by less than one percent over the wavelength range from 750 to 1100 nm. This reproducibility is shown with three replicate scans in the same color in the figures. Cow blood, which had low [Hb] and high acidity, yielded spectra similar to that of human blood of normal pH and normal hemoglobin.

A clinician using the SCiO device as a screening tool for carbon monoxide poisoning needs to know if the device can accurately detect clinically significant increases in blood carbon monoxide levels. Using a logistic fit model and a receiver operating characteristic (ROC) curve analysis, we found that the area under the ROC curve to detect ≥15% COHb using a cutoff in SCiO index value of 1.6 (from the regression equation) was 100%. Similarly, we found that the positive predictive value for calculated [COHb] with the linear model was high. For thresholds of ≥5% COHb, ≥10%COHb and ≥15% COHb, the positive predictive vale of high readings was 83–100% and the negative predictive value of values below threshold was 88–100% (Table 1). Sensitivity in detecting values greater than or equal to these ranges was 92–100%, and specificity was 62.5–100%. For the clinically important range of ≥15% COHb, sensitivity, specificity, and accuracy were all 87.5%% or greater.

**Table 1:** Predictive power of a simple linear model* derived from SCiO spectrograms to detect carboxyhemoglobin in single drops of human blood.

	5%≥COHb threshold	15%≥COHb threshold	20%≥COHb threshold
**N**	14	13	10
**PPV %** TP/(TP+FP)	83.3%	92.8%	100%
**NPV %** TN/(TN+FN)	100%	87.5%	90%
**Sensitivity** TP/(TP+FN)	100%	93.3%	92.3%
**Specificity** TN/(TN+FP)	62.5%	87.5%	100%
**Accuracy** (TP+TN)/P+N	90.9%	90.9%	95.5%

$*[COHb]=(\left(\frac{\Delta (875nm-775nm)}{925nm}\right)-1.94)/-.01982$, based on regression analysis (See Fig 2). The estimated [COHb] from this equation was compared to the measured [metHb] from the hemoximeter and scored as a true positive, true negative, false positive or false negative.

Abbreviations: PPV, positive predictive value; NPV, negative predictive value; TP, true positives; TN, true negatives; FP, false positives; FN, false negatives.

### Detection of methemoglobin

**Fig 3:**
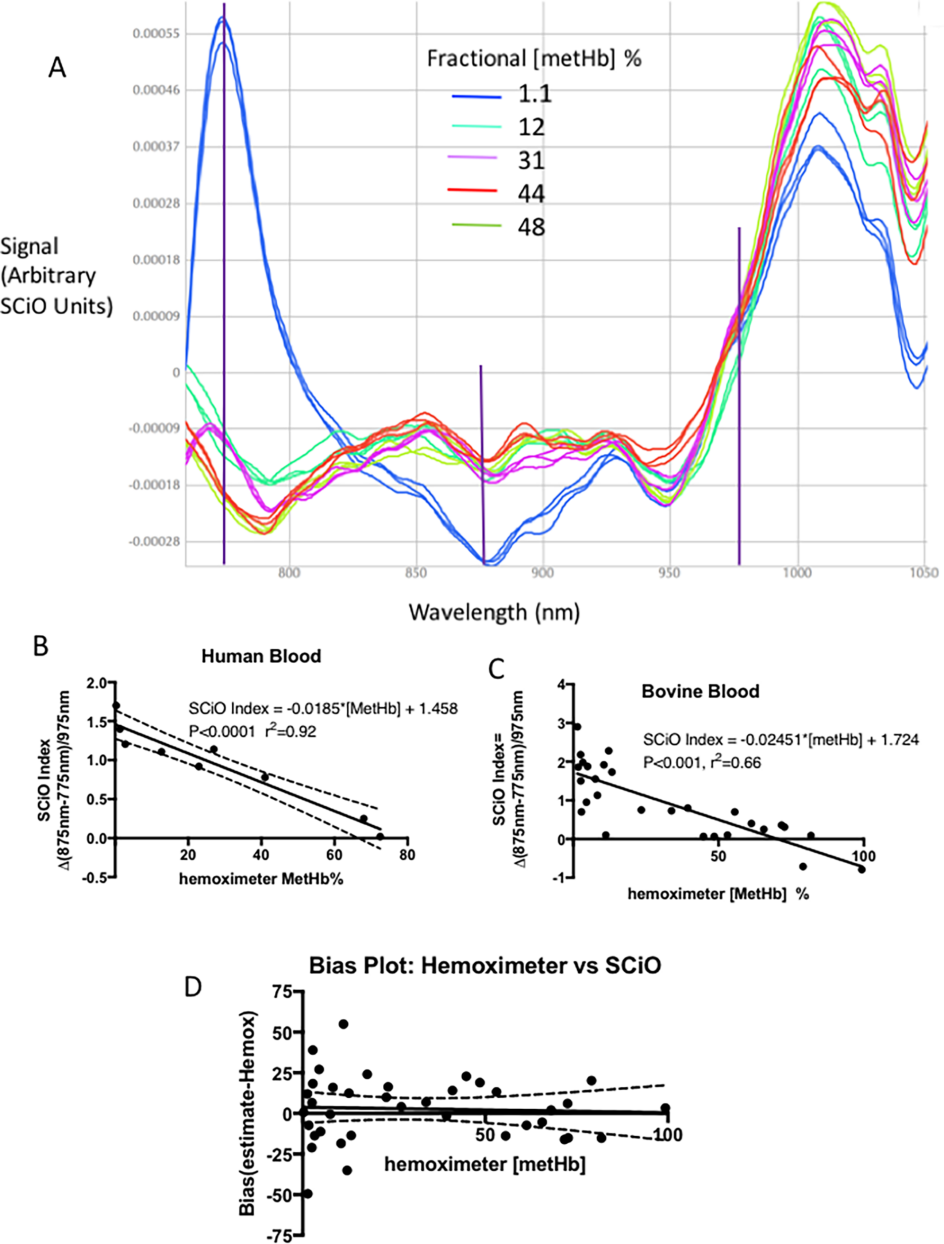
Detection of methemoglobin (MetHb) in 50 μL thin films of human and bovine blood with *in vitro* with spectrograms from the SCiO pocket molecular sensor. **A**. Representative SCiO spectrograms of human blood with increased fractional content of MetHb, produced by adding NaNO_3_ solution to syringes of heparinized blood. The wavelengths used in the spectrophotometric model (775 nm, 875 nm and the isobestic wavelength of 975 nm) are indicated by the vertical lines). **B**. Regression analysis comparing the spectrometric model and hemoximeter measured MetHb levels in human blood. **C**. Regression analysis comparing the spectrometric model and hemoximeter measured MetHb levels in bovine blood. **D**. Modified Bland-Altman bias plot of errors in SCiO estimates of [MetHb] % based on the regression equations relating SCiO MetHb Index value and hemoximeter measured [MetHb] for human and bovine blood.

Visual inspection of SCiO spectra showed changes in spectrogram signals that were [MetHb]-dependent at 750 and 800 nm, with 975 nm an isobestic wavelength (Fig 3A). A simple linear model describing the relationship between spectrogram signal at 875 nm, 775nm and 975 nm wastherefore:

$$[MetHb]=\frac{f(875nm\;spectrogram\;signal-775nm\;spectrogram\;signal)}{975nm\;signal}$$

We term the 875-775/975 ratio the “SCiO MetHb Index”. The data from this analysis are plotted as scattergrams with regression lines in Fig 3B (human blood) and 3 C (bovine blood). A modified Bland-Altman bias plot of errors in [MetHb] predicted by the SCiO scanner is shown in Fig 3D. The mean bias of this estimated [MetHb] was 2.68%, the precision (standard deviation of the bias) was 21.1% and the RMS error was 20.8%.

Clinicians using the SCiO as a screening tool for methemoglobinemia need to know the probability of detecting increases in [MetHb] above certain clinically relevant levels or thresholds. The levels at which humans become symptomatic with MetHb depend both on the MetHb level and on how long the MetHb has been present; individuals with genetic defects in MetHb metabolism may tolerate >30% MetHb without symptoms except decreased exercise tolerance, whereas a sudden increase in MetHb in an elderly person with coronary artery disease may be life-threatening if [MetHb] reaches 20%. Based on studies in humans in which MetHb was purposely increased by administration of nitrates, an acute increase in MetHb in excess of 15% typically will cause low blood pressure (especially postural hypotension) and headache. More severe symptoms are present above 20%[11]. Using a logistic fit model and a receiver operating characteristic (ROC) curve analysis, we found that the area under the ROC curve to detect ≥20% MetHb was 86%. For thresholds of ≥10% MetHb, ≥20%MetHb and ≥30% MetHb, the positive predictive value of high readings was 67–80% and the negative predictive value of values below threshold was 73–100% (Table 2). Sensitivity in detecting values greater than or equal to these ranges was 85–100%, and specificity was 57–79%. For the clinically important range of ≥20% MetHb, sensitivity, specificity, and accuracy were all 71% or greater.

**Table 2:** Predictive power of a linear model* based on SCiO spectrograms to detect methemoglobin in single drops of human and bovine blood.

	10%≥metHb threshold	20%≥metHb threshold	30%≥meHb threshold
**N**	21	15	12
**PPV %** TP/(TP+FP)	77.3%	66.7%	80%
**NPV %** TN/(TN+FN)	72.7%	100%	100%
**Sensitivity** TP/(TP+FN)	85%	100%	100%
**Specificity** TN/(TN+FP)	57.1%	70.6%	77.8%
**Accuracy** (TP+TN)/P+N	73.5%	73.5%	88.2%

$*[metHb]==(\left(\frac{\Delta (875nm-775nm)}{925nm}\right)-1.724)/-.01982$, based on regression analysis (See Fig 3). The estimated [metHb] from this equation was then compared to the measured [metHb] from the hemoximeter and scored as a true positive, true negative, false positive or false negative.

Abbreviations: PPV, positive predictive value; NPV, negative predictive value; TP, true positives; TN, true negatives; FP, false positives; FN, false negatives.

## Discussion

The results of this study show that a relatively inexpensive pocket-sized consumer infrared scanner and smart phone are capable of detecting clinically significant increases in carboxyhemoglobin and methemoglobin in a 50μL drop of blood. With further development, devices like the SCiO could be used to reduce the barriers to making critical diagnoses in resource-limited settings. The SCiO device is accurate at detecting carboxyhemoglobin (sensitivity and specificity for detecting [COHb] >15% is >88%) but somewhat less accurate for methemoglobin. Therefore, the method is a potentially valuable screening tool for detecting life-threatening levels of COHb or MetHb. However, the method we describe currently lacks the precision of laboratory CO-oximeters or pulse CO-oximeters cabable of assessing COHb and MetHb levels in blood or non-invasively through the skin[11].

We are not aware of any previous efforts to devise a simple screening tool for the presence of life-threatening levels of COHb. This is significant given the number of individuals affected by carbon monoxide poisoning each year world-wide. Previous effors to evaluate inexpensive methods to assess metHb have been based on difficult to quantify color differences in blood. These methods have involved placing blood on filter paper and imaging with a flat-bed scanner or comparing the color of blood placed on filter paper to a color chart [20–22]. The accuracy and performance of the methods, as well as the effect of total Hb and pH variations on these methods, were defined in a more limited way than presented here. The sensitivity and specificity of these other assays were not reported.

Dyshemoglobinemias are not uncommon conditions in developing countries. The most common life threatening dyshemoglobinemia is caused by carbon monoxide (CO) poisoning. CO poisoning kills more than 40,000 people each year and sickens many hundreds of thousands. China estimates 10,000 deaths per year from CO poisoning and in the US the number is 1000 per year[2, 4]. The diagnosis can be missed, because CO poisoning mimics the flu, with mailaise, nausea, headache, and weakness. Methemoglobinemia is also often a missed diagnosis in the US and other developed countries, because even with the availability of a CO-oximeter, a clinician must first suspect that a dyshemoglobin is present. MetHb is relatively common in exposures to nitrates such as the meat packing, fertilizer and chemical industry and a simple screening tool for methemoglobin could be of significant value. The incidence of methemoglobinemia is not known with certainty. Numerous case reports document the life threatening effects of acute methemoglobinemia[23].

### What levels of COHb and MetHb are clinically concerning?

Spectrograms revealed clear differences between blood containing normal and elevated levels of both COHb and metHb. Based on the scans, we created a simple linear models that related reflectance light intensity versus concentration. A receiver operating characteristic analysis showed that the ability to detect COHb or MetHb levels of 15% or greater was >87% (Tables 1 and 2). We believe that with a minimum amount of additional model building, that this accuracy could be increased to at least 90%. The cutoff levels of 15 or 20% are based on observations that in healthy subjects, metHb and COHb levels <15% do not produce clinical symptoms. 15% COHb has been found in chain smokers and garage workers[3, 24]. Although the threshold for clinical illness must vary with age and co-existing disese, 15% COHb and 20% metHb seems like a threshold of concern for all individuals.

We found that simple linear models of spectrogram analysis showed 87.5–100% sensitivity in detecting >15% COHb and >20% MetHb in both bovine and human blood. Because all vertebrate hemoglobins have similar optical properties[25], the species similarity was expected. The methods are relatively insensitive to total [Hb] and to blood pH because similar performance of the SCiO was seen in cow and human blood. The bovine blood was had a Hb and a low pH, conditions that are found in clinical samples from developing countries, where anemia is common and acidosis is a result of decreased blood oxygen delivery caused by the dyshemoglobinemia. These results suggest that our method is relatively robust and would not be affected by differences in total hemoglobin concentration or blood chemistry differences.

### Limitations of the SCiO in diagnosing CO poisoning or methemoglobinemia

The SCiO clearly underperforms in terms of the accuracy of MetHb and COHb detection compared to pulse co-oximeters such as the Masimo Rainbow and clinical CO-oximeters, which are accurate to within 1–3%. With RMS errors for MetHb and COHb of ca. 20%, we are not suggesting that the SCiO scanner can replace these other instruments for accurate measurement of blood COHb or MetHb levels. However, as a simple screening tool in situations of clinical concern, we argue that, if there are no other available assays, the SCiO provides useful information to increase the suspicion for a diagnosis of CO poisoning or methemoglobinemia. If methemoglobinemia is suspected, treatment with intravenous methylene blue can reduce MetHb levels[23]. CO poisoning is treated with oxygen, and if severe symptoms are present, hyperbaric oxygen treatment is indicated. In resource-poor areas, hyperbaric oxygen is probably not available.

Although we found that severe acidosis and anemia (bovine blood) did not interfere with COHb and metHb estimated from SCiO scans (Figs 2 and 3), we did not determine if other substances or conditions affect accuracy. For this reason, a suspected COHb or metHb diagnosis based on screening with the SCiO device should be confirmed, whenever possible,with CO-oximetry.

This study was based on blood samples. It seems possible that the SCiO could be used to diagnose carbon monoxide poisoning or the presence of methemoglobin by scanning through the human skin rather than a drop of blood. This is currently under investigation in our laboratory.

## Conclusions

We have found that a pocket infrared scanner and a smartphone is potentially a useful screening tool for detecting clinically significant increases in COHb and MetHb in a drop of blood. For the clinically important range of >15% COHb and >20% metHb, a simple calculation based on instrument readings was 94–100% sensitive and 71–88% specific in detecting these dyshemoglobins. With some refinement, the SCiO and similar devices may provide access to diagnostic information usually inaccessible in resource-poor regions.
